# Examining the correlates of cigarette smoking, e-cigarette use and dual use among Canadian post-secondary students

**DOI:** 10.1177/1179173X241247414

**Published:** 2024-04-15

**Authors:** Matthew James Fagan, Jian Kun Zhan, Kelly B. Wunderlich, Guy Faulkner

**Affiliations:** School of Kinesiology, 8166 University of British Columbia, Vancouver, Canada

**Keywords:** E-Cigarette, cigarette, nicotine, post-secondary students, dual use

## Abstract

Many Canadians use nicotine products such as cigarettes and e-cigarettes. A particular subpopulation of concern is post-secondary students given they have a higher prevalence of use. Many correlates of cigarette smoking and e-cigarette use have been identified. However, less focus has been on examining the correlates of cigarette smoking, e-cigarette use and dual use. This study explores the correlates of different nicotine modality use in post-secondary students. Using data from the Canadian Campus Wellbeing Survey (CCWS; *n* = 27,164), a multi-level nominal regression assessed the correlates of nicotine modality use. In comparison to individuals who were <20, individuals 20-24 (OR = .448, 95% CI .321, .625), 25-29 (OR = .140, 95% CI .093, .212), 30-34 (OR = .076, 95% CI .046, .125) and over 35 (OR = .041, 95% CI .024, .071) had lower odds of e-cigarette use compared to cigarette smoking. Identifying as a woman (OR = 1.553, 95% CI 1.202, 2.006), non-heterosexual (OR = .642, 95% CI = .485,0.851), current cannabis user (OR = 1.651, 95% CI 1.296, 2.104), and being an international student (OR = .350, 95% CI .251, .487) also impacted the odds of e-cigarette use vs only cigarette smoking. When considering dual use vs cigarette smoking, individuals aged 20-24 (OR = .491, 95% CI .337, .717), 25-29 (OR = .221, 95% CI .137, .357), 30-34 (OR = .163, 95% CI .091, .292) and over 35 (OR = .122, 95% CI .065, .230) had lower odds than individuals <20. Current cannabis use (OR = 1.680, 95% CI = 1.209, 2.138), binge drinking (OR = 1.885, 95% CI 1.384, 2.568), and international student status (OR = .689, 95% CI .476, .996) also impacted cigarette smoking vs dual-use. Overall, a minority of young adults (11.5%) at post-secondary institutions in our sample use nicotine products, and the higher prevalence of e-cigarette use warrants continued monitoring. Health promotion campaigns addressing e-cigarette use are required. Additionally, tailored intervention efforts could prioritize the treatment needs of international students studying in Canada.

## Introduction

Nicotine, a highly addictive and hazardous psychoactive substance, can have detrimental effects on various organs in the human body.^1^ Unsurprisingly, tobacco consumption, a primary source of nicotine, continues to contribute significantly to global mortality and morbidity rates.^2^ In Canada, cigarette smoking and e-cigarette use are the predominant modes of nicotine consumption.^3^ According to Statistics Canada,^4^ the number of individuals smoking cigarettes aged 12 years and older decreased from 5.3 million in 2015 to 3.8 million in 2021. However, there has been a significant surge in e-cigarette usage in recent years. Globally, e-cigarette users rose from 7 million in 2011 to 41 million in 2018.^5^ E-cigarette use is particularly prevalent among Canadian youth and young adults.^6^ In 2021, nearly half (48%) of young adults aged 20-24 and approximately 29% of youth aged 15-19 reported trying e-cigarettes, compared to only 13% of adults over the age of 25^6^.

E-cigarettes are often promoted as a safer alternative to smoking and a potential tool for smoking cessation.^7^ Additionally, research has indicated that young adults (aged 20-24) primarily use e-cigarettes for enjoyment (27%), stress reduction (25%), and smoking cessation (24%).^6^ However, there are concerns that e-cigarette use may lead to smoking initiation.^8^ Studies have shown that e-cigarette users have 2 to 4 times higher odds of expressing an intention to smoke cigarettes compared to non-e-cigarette users.^9,10^ It is important to note that the relationship between e-cigarette use and smoking initiation is likely influenced by age.^2^ For example, Soneji et al^11^ found that adolescents who reported using e-cigarettes had more than six times higher odds of starting to smoke cigarettes than those who had never used e-cigarettes. Understanding factors related to dual use of e-cigarettes and cigarettes compared to using only one modality would provide potential avenues for prevention and intervention as there is currently no agreement on a recommended general approach for the treatment of people who are both smoking and vaping and are seeking help to quit.^12^

Several studies have explored the factors associated with cigarette smoking or e-cigarette use.^13-16^ For instance, demographics (such as age, mental health, sex, gender, and sexuality), health behaviours (including physical activity and nutrition), and institutional factors (such as school closures due to COVID-19) have been identified as correlates of smoking.^13-16^ Many of these factors also play a role in e-cigarette use.^13-18^ However, there have been conflicting findings regarding the correlates of e-cigarette use and smoking. For example, sport participation has been positively associated with e-cigarette use but negatively associated with smoking among secondary students.^15^ Further research is needed to understand the factors associated with the dual use of e-cigarettes and cigarette smoking. There is limited work that has attempted to identify the correlates of dual use of cigarettes and e-cigarettes.^19-21^ One study found that depressive symptoms and adverse childhood experiences (ACEs) were associated with all trajectories of dual use.^19^ Piper et al. found that dual users were more likely to be younger and white and did not differ in their intentions to quit smoking.^20^ Finally, Zavala-Arciniega et al, found that in a Mexican sample, compared to individuals only smoking cigarettes, dual users were more likely to be younger, have a higher smoking dependence, and recently quit smoking.^21^

Understanding the factors contributing to different nicotine use patterns among post-secondary students could inform targeted interventions for educational institutions. These interventions could consider demographic factors such as gender, age, education level, and socioeconomic status. Additionally, it is important to consider health-related factors such as alcohol consumption and physical activity levels. There is also limited literature on the dual use of e-cigarettes and cigarettes compared to the singular use of either product among post-secondary students. This study explores the factors associated with the dual use of cigarettes and e-cigarettes vs cigarettes alone.

## Methods

### Data collection

This study uses data from the 2022 Winter deployment of the Canadian Campus Wellbeing Survey (CCWS; https://www.ccws-becc.ca/). The Winter cycle included 21 post-secondary institutions across Canada with a mean response rate of 18.8%. This cycle from the CCWS has been used previously.^22^ Please see Faulkner et al.^23^ for further description of the CCWS. This project was approved by the appropriate institutional ethics board (approval H19-01907) and the CCWS Data Access Committee approved using aggregated data.

## Measures

### Outcome measure

Modality of nicotine use was the primary outcome of this study. This includes three categories: smoking cigarettes, e-cigarette user or dual user. Cigarette smoking was collected in the CCWS by asking, “Which of the following best applies to you?” with response options ranging from “*I smoke cigarettes every day”* to “*I have never been a smoker*”. Consistent with previous work,^22^ a dichotomous measure of currently smoking cigarettes was created by including “*I smoke cigarettes every da*y” and “*I smoke cigarettes but not every day*” as current use. This was done as current use is typically defined as use within the last month.^24^

The CCWS collects e-cigarette use by asking, “*During the past 30 days, how often did you use an e-cigarette or vaping device*?” with the response options ranging from *daily or almost daily to never*. Consistent with previous work,^22^ a dichotomous variable was created to reflect current use.^24^ Finally, if an individual was found to use cigarettes and e-cigarettes through these measures, they were coded as dual users. It should be noted that the response options for both cigarette use and e-cigarette use include “*I don’t know*” and “*I prefer not to answer*”. If an individual selected one of these options for either cigarette smoking or e-cigarette use, they were excluded from the analysis.

### Predictors

#### Demographic and institutional-level factors

Consistent with previous work,^22^ several demographic covariates were used in this analysis. These demographic covariates included; age groups (*>20 (ref),20-24, 25-29, 30-34, ≥35 years old*), gender (*man (ref), woman, non-binary person, two-spirit, I prefer not to answer*), ancestry (*White (ref), Asian, Indigenous, other*), trans experience (n*o (ref), yes*), sexual orientation (*heterosexual (ref), non-heterosexual, I prefer not to answer*), and international student status (*domestic (ref) or international*).

Additionally, food security was used as a proxy for socioeconomic status in our study. It was assessed through six items from the Canadian Community Health Survey; Household Food Security Survey Module.^25^ Each item asks about food security in the last 12 months. Each question was coded affirmative or negative based on the response, scoring 1 for affirmative and 0 for negative, creating an integer from 0-6. One example item is “*The food that (I/we) brought just did not last, and (I/we) did not have money to get more*”. This score is then categorized into three levels: food secure (scores <2), low food security (2-4) and very low food security (5 or greater). This measure has been used previously as a proxy for socioeconomic status with the CCWS data.^22^

Finally, institutional-level factors were included in the data analysis. More specifically, the location of the institution (*Alberta (ref), Ontario, British Columbia, Atlantic provinces, Quebec/Manitoba)*, college or university institution, and the institution size in terms of enrollment *(<5000 (ref), 5001-20000, 20001- 40000, >40000 students*).

#### Physical activity

The amount of physical activity was captured using the International Physical Activity Questionnaire (IPAQ).^26^ The IPAQ assesses weekly moderate and vigorous physical activity (MVPA) performed in bouts greater than 10 minutes. Following IPAQ truncation rules, each intensity of physical activity was capped at 180 minutes a day. This amount of MVPA was then used to create a variable to reflect meeting the Canadian MVPA guidelines of 150 MVPA minutes/week.^27^ When considering the psychometrics of the IPAQ, it has a criterion validity similar to other physical activity questionnaires (r = .30) and a high test-retest reliability (r = .80).^28^

#### Sleep

The items used to assess sleep followed evidence-based guidelines to measure self-reported sleep in Canadians through population-based surveillance systems.^29^ Sleeping and waking time were collected on a typical weekend and weekday (response options are available in 30-minute increments). These four items calculate total sleep for weekends and weekdays separately. The current study created a dichotomous variable of meeting the sleep guidelines by identifying individuals who met the sleep recommendations on both weekends and weekdays (7-9 hours;^27^).

#### Cannabis use

Cannabis use was measured by asking, “*In the past 30 days, how often did you use cannabis?*”. The response options ranged from *not in the past 30 days* to *daily*. Consistent with previous work with the CCWS,^22^ a dichotomous measure of current use of cannabis was created to indicate use in the last 30 days.^24^

#### Binge drinking

Binge drinking was measured by asking, “*During the past 30 days, how often have you had 4 or more drinks (female sex) OR 5 or more drinks (male sex) on one occasion?”*. The response options range from *daily* to *I do not drink alcohol*. Consistent with previous work with the CCWS,^22^ our analysis created a dichotomous variable of current binge drinking, to reflect binge drinking at least once in the last month.^24^

#### Mental distress

Consistent with previous work with the CCWS,^22^ the Kessler Psychological Distress Scale (K10) was used to collect a global measure of psychological distress.^30,31^ The K10 has shown acceptable reliability and validity across sociodemographic subsamples.^30^ Each item within the K10 is scored on a scale from 1-5. The ten items are then summed to obtain a total score ranging from 10 to 50. After obtaining the total score, categories were created to reflect the level of psychological distress; well (scores <19), mild mental distress (scores 20-24), moderate mental distress (scores 25-29), and severe mental distress (scores >29).

### Data analysis

Descriptive statistics were used to determine demographic and mental health differences (Student’s *t*-test for continuous variables and a chi-square test for categorical). All analyses were conducted using *R version 4.2.1* and *R Studio*. The *mblogit version .9.4.2* package was used to create a multi-level nominal regression as the students are nested within their institutions. The multi-level nature of the regression was included based on the ICC and the design effects calculated.^32^ Using the *mblogit* function, a multi-level nominal regression was created to assess the correlates of the modality of nicotine use.

### Data inspection and data exclusion

To be included in the analysis, a participant must have been classified as currently smoking cigarettes, using an e-cigarette or dual using [11.45% of the total sample (3105/27,164)]. Missing data on the included variables ranged from 0 to ∼19%. There was an indication the data were not missing at random. For example, individuals missing gender or sexual orientation were more likely to be missing other data (e.g., cigarette smoking or e-cigarette use). As the CCWS sample is not representative and data were not missing at random, our analysis only includes complete cases.^33^

## Results

### Descriptive statistics

Forty-nine percent of participants (n = 3105) identified as White, 57% as women, and 80% as domestic students. Of the 3105 nicotine users, 22%, 58%, and 20% of the sample were smoking cigarettes, e-cigarette users or dual users, respectively (see Table 1 for a breakdown of demographics by nicotine modality).

**Table 1.** Demographic and Descriptive Statistics.

Characteristic	Smoking cigarettes, N = 682^a^	E-cigarette use, N = 1,789^a^	Dual use, N = 634^a^	*P*-value^b^
Gender				
Man (ref)	286 (42%)	551 (31%)	229 (37%)	
Woman	344 (51%)	1155 (65%)	349 (56%)	
Non-binary	29 (4.3%)	51 (2.9%)	23 (3.7%)	
Two-spirit	5 (.7%)	3 (.2%)	6 (1.0%)	
Prefer not to answer	15 (2.2%)	12 (.7%)	14 (2.3%)	
Missing	3	17	13	
Trans experience				<.001
No	635 (94%)	1703 (96%)	575 (93%)	
Yes	28 (4.1%)	45 (2.5%)	23 (3.7%)	
Prefer not to answer	16 (2.4%)	21 (1.2%)	23 (3.7%)	
Missing	3	20	13	
Age group				<.001
Under 20	70 (10%)	655 (37%)	186 (30%)	
20-24	252 (37%)	889 (50%)	302 (48%)	
25-29	145 (21%)	147 (8.2%)	76 (12%)	
30-34	98 (14%)	48 (2.7%)	30 (4.8%)	
Over 35	115 (17%)	43 (2.4%)	33 (5.3%)	
Missing	2	7	7	
Sexual orientation				<.001
Heterosexual/Straight	444 (66%)	1234 (70%)	397 (64%)	
Non-heterosexual	202 (30%)	499 (28%)	194 (31%)	
I prefer not to answer	29 (4.3%)	34 (1.9%)	29 (4.7%)	
Missing	7	22	14	
Ethnicity				<.001
White	296 (43%)	1000 (56%)	285 (46%)	
Asian	214 (31%)	346 (19%)	192 (31%)	
Indigenous	27 (4.0%)	38 (2.1%)	12 (1.9%)	
Other	144 (21%)	396 (22%)	133 (21%)	
Missing	1	9	12	
Food security				.034
Food secure	286 (53%)	803 (58%)	253 (52%)	
Low food security	123 (23%)	300 (22%)	117 (24%)	
Very low food security	131 (24%)	272 (20%)	120 (24%)	
Missing	142	414	144	
Met MVPA				<.001
No	261 (38%)	491 (28%)	221 (35%)	
Yes	421 (62%)	1294 (72%)	409 (65%)	
Missing	0	4	4	
Met sleep				.10
No	349 (53%)	988 (57%)	357 (59%)	
Yes	308 (47%)	749 (43%)	249 (41%)	
Missing	25	52	28	
Current cannabis use				<.001
No	380 (56%)	663 (37%)	265 (42%)	
Yes	298 (44%)	1111 (62%)	359 (57%)	
I don’t know/Prefer not to answer	2 (.3%)	15 (.8%)	10 (1.6%)	
Missing	2	0	0	
Current binge drinking				<.001
No	261 (38%)	510 (29%)	141 (22%)	
Yes	404 (60%)	1269 (71%)	480 (76%)	
I don’t know/Prefer not to say	13 (1.9%)	10 (.6%)	13 (2.1%)	
Missing	4	0	0	
E-cigarette use				<.001
Daily or almost daily	0 (0%)	851 (48%)	352 (56%)	
Less than daily, but at least once a week	0 (0%)	394 (22%)	148 (23%)	
Less than weekly, but at least once in the past 30 days	0 (0%)	544 (30%)	134 (21%)	
Never	495 (73%)	0 (0%)	0 (0%)	
Not in the past 30 days, but from time to time	187 (27%)	0 (0%)	0 (0%)	
Tobacco use				<.001
I do not smoke cigarettes at all, but I do smoke tobacco of some kind (e.g. Pipe or shisha)	0 (0%)	301 (17%)	0 (0%)	
I have never been a smoker (i.e. smoked for a year or more)	0 (0%)	1175 (66%)	0 (0%)	
I have stopped smoking completely in the last year	0 (0%)	131 (7.3%)	0 (0%)	
I smoke cigarettes (including hand-rolled) every day	283 (41%)	0 (0%)	208 (33%)	
I smoke cigarettes (including hand-rolled), but not every day	399 (59%)	0 (0%)	426 (67%)	
I stopped smoking completely more than a year ago	0 (0%)	182 (10%)	0 (0%)	
Mental distress				<.001
Well	111 (16%)	193 (11%)	58 (9.3%)	
Mild mental distress	123 (18%)	322 (18%)	102 (16%)	
Moderate mental distress	143 (21%)	350 (20%)	125 (20%)	
Severe mental distress	298 (44%)	907 (51%)	341 (54%)	
Missing	7	17	8	
Province				<.001
Alberta	55 (8.1%)	169 (9.4%)	53 (8.4%)	
Atlantic	15 (2.2%)	117 (6.5%)	36 (5.7%)	
British Columbia	84 (12%)	228 (13%)	82 (13%)	
Manitoba/Quebec	92 (13%)	260 (15%)	71 (11%)	
Ontario	436 (64%)	1015 (57%)	392 (62%)	
Student status				<.001
Domestic	487 (71%)	1604 (90%)	495 (78%)	
International	195 (29%)	185 (10%)	139 (22%)	
Size of institution				<.001
<=5000	30 (4.4%)	97 (5.4%)	26 (4.1%)	
5001-20000	161 (24%)	523 (29%)	141 (22%)	
20001-40000	181 (27%)	556 (31%)	174 (27%)	
>40000	310 (45%)	613 (34%)	293 (46%)	

^a^n (%).

^b^Pearson’s Chi-squared test.

### Correlates of nicotine modality

Several correlates of nicotine modality were identified in our analyses. For ease of interpretation from the multi-level nominal regression, a breakdown of individuals smoking cigarettes vs e-cigarette users will be presented, followed by individuals smoking cigarettes vs dual users (see Table 2).

**Table 2.** Results From the Multi-Nominal Regression.

Smoking Cigarettes (ref) Vs e-Cigarette User			Smoking Cigarettes (ref) Vs Dual Use	
	OR	95%CI	OR	95%CI
Gender				
Man (ref)	-	-	-	-
Woman	**1.553**	**1.202, 2.006**	1.194	.885, 1.611
Non-binary	1.212	.552, 2.662	.769	.312, 1.895
Two-spirit	.713	.119, 4.265	2.172	.406, 11.607
Prefer not to answer	.444	.223, 1.889	.649	.152, 1.295
Trans experience				
No	**-**	-	-	-
Yes	**.**654	.305, 1.403	.974	.416, 2.279
Prefer not to answer	**.**783	.297, 2.063	1.502	.574, 3.928
Age group				
Under 20	**-**	-	-	-
20-24	**.448**	**.321, .625**	**.491**	**.337, .717**
25-29	**.140**	**.093, .212**	**.221**	**.137, .357**
30-34	**.076**	**.046, .125**	**.163**	**.091, .292**
Over 35	**.041**	**.024, .071**	**.122**	**.065, .230**
Sexual orientation				
Heterosexual	**-**	-	-	-
Non-heterosexual	**.642**	**.485, .851**	.916	.662, 1.267
Prefer not to say	.998	.474, 2.099	1.356	.609, 3.016
Ethnicity				
White	**-**	-	-	-
Asian	.965	.694, 1.343	1.302	.892, 1.901
Indigenous	.711	.338, 1.494	.728	.284, 1.866
Other	1.372	.999, 1.885	1.126	.775, 1.637
Food security				
Food secure	**-**	-	-	-
Low food security	**.**930	.688, 1.257	1.031	.726, 1.464
Very low food security	.781	.575, 1.061	1.003	.706, 1.425
Met MVPA				
No	**-**	-	-	-
Yes	1.141	.888, 1.464	.952	.713, 1.270
Met sleep				
No	**-**	-	-	-
Yes	1.006	.793, 1.277	.830	.629, 1.097
Current cannabis use				
No	**-**	-	-	-
Yes	**1.651**	**1.296, 2.104**	**1.608**	**1.209, 2.138**
I don’t know/Prefer not to answer	2.831	.602, 13.311	4.457	.887, 22.394
Current binge drinking				
No	**-**	-	-	-
Yes	1.153	.895, 1.484	**1.885**	**1.384, 2.568**
I don’t know/Prefer not to say	.562	.174, 1.808	1.779	.597, 5.305
Mental distress				
Well	**-**	-	-	-
Mild mental distress	**.**807	.525, 1.241	1.027	.593, 1.780
Moderate mental distress	.865	.564, 1.326	1.304	.763, 2.228
Severe mental distress	.999	.672, 1.486	1.638	.992, 2.704
College or university				
College	**-**	-	-	-
University	**.**963	.557, 1.665	1.139	.565, 2.299
Province				
Alberta	**-**	-	-	-
Atlantic	.982	.312, 3.093	.570	.148, 2.195
British Columbia	.738	.293, 1.859	.717	.242, 2.120
Manitoba/Quebec	**.**611	.262, 1.427	.446	.166, 1.195
Ontario	**.**628	.310, 1.273	**.396**	**.180, .873**
Student status				
Domestic	**-**	-	-	-
International	.**350**	**.251, .487**	**.689**	**.476, .996**
Size of institution				
<=5000	**-**	-	-	-
5001-20000	.943	.399, 2.228	1.218	.419, 3.542
20001-40000	1.241	.559, 2.756	1.149	.441, 2.991
>40000	.881	.368, 2.104	1.451	.505, 4.168

Notes: OR = Odds ratio; CI = confidence interval; Bold OR and 95%CI = *P* < .005.

### Smoking cigarettes vs e-cigarette users

Compared to men, women had 55% higher odds of reporting current e-cigarette use than smoking. Compared to <20 years, individuals 20-24, 25-29, 30-34 or over 35 years old had approximately 65%, 86%, 93% and 96% lower odds of being an e-cigarette user, respectively. Compared to individuals who identified as heterosexual, non-heterosexuals were at 38% lower odds of being an e-cigarette user than smoking cigarettes. Individuals who were current cannabis users had 65% higher odds of being an e-cigarette user compared to smoking cigarettes. Finally, international students were at 65% lower odds of being e-cigarette users when compared to smoking cigarettes.

### Smoking cigarettes vs dual-users

Compared to <20 years, individuals 20-24, 25-29, 30-34 or over 35 years old had approximately 51%, 78%, 84%, and 88% lower odds of being a dual-user, respectively. Individuals who were current cannabis users were at 61% higher odds of being a dual user than smoking cigarettes. Individuals who were current binge drinkers had 89% higher odds of being a dual user compared to smoking cigarettes. Individuals attending post-secondary institutions in Ontario were at 60% lower odds of being dual users when compared to smoking cigarettes. Finally, international students were at 31% lower odds of being dual users when compared to smoking cigarettes.

## Discussion

This paper aims to provide insights into the factors associated with the modality of nicotine use in a large sample of Canadian post-secondary students. Given that nicotine use, particularly cigarette smoking, remains a leading cause of morbidity and economic burden in Canada,^34,35^ it is crucial to gain a better understanding of usage patterns. Our study found approximately 11.5% of the total sample were using nicotine. Of the nicotine users, 22% were currently smoking cigarettes, 58% were current e-cigarette users, and 20% were dual users. A similar prevalence has been found in the Canadian Tobacco and Nicotine Survey (CTNS) in 2020, as the prevalence of smoking in young adults (age 20-24) was approximately 8% and 11% among adults (older than 25).^36^ However, in a post-secondary context, the Canadian Post-secondary Education Alcohol and Drug Use Survey (CPDS) found a higher prevalence of daily and occasional cigarette smoking (10%) than that of current e-cigarette users (∼6%).^37^ Broadly, these findings indicate that a minority of Canadians smoke or vape, yet continued tobacco control interventions are required.

Several demographic and health behaviours were associated with the odds of different nicotine modalities. Regarding the factors associated with cigarette smoking and e-cigarette use, several factors played a significant role. Women were more likely to use e-cigarettes than to smoke cigarettes. Our gender finding aligns with the results of the CTNS, which showed a higher prevalence of smoking among men compared to women.^6^ Additionally, participants in the older age categories were more likely to smoke cigarettes rather than use e-cigarettes, consistent with the CTNS findings.^6^ However, the association of age with modality should be examined longitudinally, considering recent regulations implemented to deter youth from using e-cigarettes.^38^

Regarding substance use, current cannabis use was associated with increased odds of e-cigarette use compared to cigarette smoking. Other work has found cannabis use to be positively associated with e-cigarette use among adolescents and young adults.^39^ However, it has also been associated with cigarette smoking.^40^ A possible explanation for our finding is the potential that young adults are choosing to use electronic devices for the consumption of cannabis (vaporizers), leading young adults to consume nicotine similarly. However, our results cannot directly confirm this possibility. Future work could assess this hypothesis.

Our study contributes to the existing literature by identifying that individuals who identify as non-heterosexual have higher odds of using cigarettes compared to e-cigarettes. Previous studies have identified that members of the 2SLGBTQ + community are at higher risk of smoking and e-cigarette use.^41–43^ However, the differentiation of modality of use in our sample could help inform targeted approaches for diverse populations to reduce nicotine use. Further research is needed to confirm this association. Lastly, it was found that international students had lower odds of using e-cigarettes compared to smoking cigarettes. Limited evidence exists on the association of international student status and smoking or vaping although certain countries have a higher prevalence of cigarette smoking than Canada.^44^ One study showed that international students exhibited higher noncompliance with smoke-free policies at a large university.^45^ This finding should be particularly relevant to post-secondary institutions with a significant number of international students, as targeted smoking cessation programs may be needed to address rates of use.

When considering factors associated with the odds of cigarette smoking vs dual use, many factors were similar to those associated with cigarette smoking vs e-cigarette use. For instance, being an older student and an international student were associated with lower odds of being a dual user. Additionally, cannabis use was found to increase the odds of dual use. However, one factor that did not differentiate cigarette smoking vs e-cigarette use but did for dual use was binge drinking. Specifically, current binge drinking was associated with higher odds of dual use compared to cigarette smoking. A recent systematic review and meta-analysis found a positive association between e-cigarette use and alcohol use, including binge drinking, among adolescents.^46^

Furthermore, alcohol use and cigarette smoking have been found to be positively associated, and alcohol consumption has been negatively associated with smoking cessation.^47,48^ However, it is interesting to note that binge drinking did not emerge as a significant factor when considering cigarettes vs e-cigarettes. Speculatively, one possible explanation could be the concept of a “social user”^49^ for whom behaviour is triggered by alcohol use. The introduction and popularity of e-cigarettes may have created a phenomenon where individuals who binge drink are susceptible to consuming various nicotine products in social settings.

Several strengths and limitations of this study should be acknowledged. Firstly, our study provides insights into factors associated with the dual use of cigarettes and e-cigarettes among at-risk populations. Secondly, we utilized a large sample of post-secondary students across Canada. However, the CCWS is a cross-sectional data surveillance system, and therefore, causality cannot be assumed. The overall response rate for the CCWS is relatively low and could introduce bias to our results. Additionally, the sample collected may not be representative of the Canadian post-secondary population. Therefore, caution is necessary when generalizing the results. Using self-reported measures in the study introduces various types of bias (e.g., desirability bias). While self-reported data on cigarette smoking status has been reported as providing a valid estimate of smoking prevalence in Canada,^50^ it may be underestimated in this sample, given reporting biases for socially undesirable behaviours.^51^ Finally, the item used to collect vaping “*During the past 30 days, how often did you use an e-cigarette or vaping device?”* does not explicitly mention that the e-cigarette or vaping device contains nicotine.

Our results have implications for policymakers and decision-makers at post-secondary institutions. Firstly, e-cigarette use is the preferred nicotine delivery method among young adults, making campaigns addressing the harms of this behaviour important.^52^ Secondly, international students may be a specific population that could benefit from cigarette cessation interventions. Targeting this subgroup may be the most effective way to reduce the harm associated with cigarette smoking in the post-secondary context in Canada. Lastly, further research is needed to understand the temporal nature of the dual-use category, as it is unclear from our study whether individuals initiated cigarette smoking or e-cigarette use and then transitioned to the other modality.

In conclusion, our findings indicate e-cigarette use is the most common modality for nicotine consumption among post-secondary students. Continued monitoring and health campaigns addressing the harms of e-cigarette use may be a starting point for post-secondary institutions to consider. Moreover, international students may require specific attention regarding cigarette smoking, and smoking cessation efforts could be tailored to this sub-population in Canada. Further research is needed to understand the dynamics of dual use and the transition between modalities.
